# Analysis of Behavioral, Growth and Metabolic Indicators in Suckling Calves Under Outdoor Winter Rearing Conditions Using Fuzzy Comprehensive Evaluation

**DOI:** 10.3390/ani16050716

**Published:** 2026-02-25

**Authors:** Jiachen Qu, Xiaojing Zhou, Jintao Liu, Zhaoyu Han, Yongli Qu

**Affiliations:** 1 Department of Animal Science, Nanjing Agricultural University, Nanjing 210018, China; qjc990313@163.com; 2 The Key Laboratory of Green and Low-Carbon Agriculture in the Northeast Plain, Department of Information and Computing Science, Heilongjiang Bayi Agricultural University, Ministry of Agriculture and Rural Affairs, No. 5 Xinyang Road, Daqing 163319, China; zhouxiaojing7924@126.com; 3 Lindian Youran Animal Husbandry Co., Ltd., Daqing 166300, China; ljtljt2026@163.com

**Keywords:** Holstein dairy calves, cold stress, blood metabolome, model, physiology, growth performance

## Abstract

This study aimed to scientifically assess cold stress in dairy calves and optimize winter rearing protocols. A combined approach of feeding trials, expert surveys, and multidimensional data analysis was used to evaluate the effects of outdoor (−5~−28 °C) and indoor (5 °C) environments on Holstein dairy calves. A 60-day controlled trial was conducted with 20 healthy 5-day-old calves. In parallel, an interdisciplinary panel of 20 experts and 8 farmers established a cold stress evaluation system via the analytic hierarchy process (AHP), with cold stress levels quantified through fuzzy comprehensive evaluation. Environmental (weight = 0.62), physiological (weight = 0.22), and behavioral (weight = 0.16) factors contributed differentially to cold stress assessment, with data showing that outdoor calves were under mild cold stress (maximum membership degree = 0.64). The temperature–humidity index (THI) showed significant correlations with multiple physiological and biochemical parameters. Generalized linear mixed model (GLMM) analysis confirmed that THI variation significantly influenced calf standing time, respiratory rate (RR), malondialdehyde (MDA) content, and total antioxidant capacity (T-AOC). In feeding trials, indoor calves exhibited marginally higher average daily gain and body weight in early stages, whereas outdoor calves demonstrated significantly better growth performance by day 60. The outdoor group displayed increased lying and defecation behaviors, along with reduced locomotor/standing time and respiratory frequency. No significant intergroup differences were observed in serum immune or antioxidant indicators. Metabolomic analysis identified 20 differentially expressed metabolites, indicating an enhancement in the activity of energy metabolism pathways in calves. This study establishes a quantitative methodology for cold stress evaluation, clarifies environment–physiology–behavior interactions, and provides a theoretical basis for winter calf management. The results confirm that outdoor cold exposure did not hinder calf growth without compromising health, offering scientific support for optimizing outdoor rearing strategies in cold regions.

## Simple Summary

This study explored winter rearing for Holstein calves via a 60-day trial (indoor 5 °C vs. outdoor −5~−28 °C). An AHP-FCE cold stress evaluation system was established, identifying outdoor calves in “mild cold stress”. During the 60-day trial period, outdoor-housed calves exhibited superior long-term growth performance and adaptive behaviors, with no adverse health effects observed, and 20 differential metabolites associated with energy metabolism were identified. The research supports optimized winter outdoor rearing in cold regions.

## 1. Introduction

China’s dairy farming industry is predominantly concentrated in northern regions such as Inner Mongolia and Heilongjiang, among which, Heilongjiang province has long ranked among the top three nationally in terms of dairy cow inventory and milk production [1], owing to its significant advantages in these areas. The region experiences prolonged and severe winters, where persistently low temperatures induce cold stress, a core challenge constraining the development of animal husbandry. When environmental temperatures fall outside the thermoneutral zone for livestock, it triggers a series of stress responses, including alterations in respiratory rate, fluctuations in body temperature, hormonal imbalances, and metabolic disturbances [2,3]. These physiological changes ultimately lead to reduced production performance and increased disease incidence [4].

Existing research confirms that cold stress significantly impacts the production performance and health of various livestock species, including growth retardation in piglets, declines in production performance and survival rates in broilers, imbalance of the antioxidant system in sheep, and suppressed immune function in Sanhe cattle [5]. To cope with low-temperature environments, homeothermic animals maintain body temperature homeostasis by initiating two thermogenic modes, shivering and non-shivering thermogenesis. Among these, non-shivering thermogenesis, mediated by the sympathetic nervous system, represents a more efficient and sustained adaptive thermogenic pathway. Its core mechanism involves the transmission of cold signals from cutaneous thermoreceptors to the hypothalamic thermoregulatory center, which subsequently activates the release of norepinephrine from sympathetic nerve terminals. Norepinephrine binds to β3-adrenergic receptors on the surface of target cells such as brown adipose tissue, triggering the cAMP/PKA signaling pathway [6]. This pathway, on one hand, activates hormone-sensitive lipase to mobilize lipolysis, and on the other hand, induces the expression and functional activation of mitochondrial uncoupling protein 1, ultimately converting chemical energy directly into heat. In northern China, insulation measures commonly implemented in livestock barns often result in inadequate ventilation, predisposing animals to respiratory and digestive diseases and creating a vicious cycle of “insulation-disease”. Although the outdoor calf hutch system has attracted attention due to its cost-effectiveness, its applicability under cold conditions still lacks systematic investigation. A previous study on Azikheli buffalo calves demonstrated how improving the quality of diet, such as using urea-molasses-treated straw, can significantly enhance dry matter intake and growth performance [7].

Regarding cold stress assessment, multidimensional evaluation indices have been established. Beyond the modified Temperature–Humidity Index (THI) criteria for cold stress classification [8], the Wind Chill Temperature (WCT) index [9], jointly proposed by the U.S. and Canadian meteorological centers, provides a more accurate assessment of cold tolerance in humans and livestock. The fuzzy comprehensive evaluation (FCE) method, grounded in fuzzy mathematics, is suitable for multidimensional comprehensive analysis. When integrated with the analytic hierarchy process (AHP) [10], it allows for scientifically determining indicator weights.

To explore whether calves can grow in the cold environment during winter and what impacts the outdoor cold environment exerts on their growth performance, immune indicators and behavioral indicators, this study innovatively integrates several external environmental factors (wind, humidity, and temperature) with behavioral and internal physiological indicators: (1) an AHP-based FCE model is constructed to assess cold stress in calves; (2) behavioral patterns, growth performance and blood parameters of suckling calves reared outdoors in cold environments versus indoors under normal temperatures are systematically analyzed. The findings aim to provide a scientific basis and practical guidance for implementing outdoor rearing systems for calves during winter in alpine regions.

## 2. Materials and Methods

### 2.1. Animals, Housing, and Feeding

This trial was conducted in accordance with the principles and responsibilities stipulated in the Animal Research Guidelines of Nanjing Agricultural University, and was carried out at a livestock experimental farm in Qiqihar city, Heilongjiang province, from November 2022 to March 2023. The farm is located at longitude 123.40° (N) and latitude 47.42° (E). The trial approval was issued by Nanjing Agricultural University. During the trial, the average outdoor temperature was −12.39 °C. Calves in the control group were housed in the calf barn, with an average temperature of 5 °C. The experimental barn faced north–south, with sliding windows on both north and south sides; all windows were closed in winter, the north wall had two doors, the east wall had one door, and the roof featured a double-pitch structure with composite color steel plates and solar panels.

Twenty female Chinese Holstein calves (age = 5 ± 1 days, body weight = 35.39 ± 1.57 kg) were randomly sorted using the random number table method into two groups: an indoor group and an outdoor group, with 10 calves in each group. No antibiotics or probiotics were administered prior to sampling. The experimental design included two groups: (1) The control group was defined as the indoor group (C), where calves were group-housed in pens within a calf barn. The pens measured 400 cm × 300 cm × 150 cm (length × width × height), and the overall dimensions of the calf barn were 122 m in length, 30 m in width, and 5.6 m in height. (2) The experimental group was defined as the outdoor group (T), where calves were individually housed in outdoor calf hutches with dimensions of 203 cm × 143 cm × 149 cm (length × width × height), with bedding comprising 15 cm thick rice husk and 15 cm thick *Leymus chinensis*, and equipped with an activity area of 170 cm × 150 cm × 107 cm. The indoor group had the same bedding as the outdoor group, and natural ventilation was applied throughout the experiment. Both groups were separated by iron fences to avoid cross-contamination. The bedding in the calf hutches and pens was replaced weekly, and the calf hutches and pens themselves were disinfected simultaneously. During the experiment, calves in both groups were fed fresh milk twice daily at 7:00 and 15:00; the milk was produced from the same farm and pasteurized at 70 °C for 1 h. From day 5 to day 10, the feed amount was increased to 7 L/day (3.5 L per meal). During the trial period, both outdoor and indoor groups of calves completely consumed the provided colostrum. From day 11 to the end of the experiment, the feeding amount was adjusted to 4 L per meal. All animals had free access to fresh warm water (38~40 °C). The outdoor group of calves was provided with supplemental warm water three times daily. From day 30 onward, pelleted calf starter concentrate, produced by Heilongjiang Hefeng Seed Industry Technology Co., Ltd., Harbin, Heilongjiang, China was provided for ad libitum intake, and the composition and chemical components of the starter concentrate were crude protein ≥ 20.0, crude fiber ≤ 20.0, crude ash ≤ 15.0, water ≤ 14.0, NaCl: 0.3–2.0, Ca: 0.3–2.0, P ≥ 0.3, lysine ≥ 0.6. Feeding times were maintained at 7:00 and 15:00. The trial period lasted for two months.

On days 1, 30, and 60 of the experiment, calves were weighed using an electronic scale (ZHUHENG ZH-240, Zhuheng Electronic Technology Co., Shanghai, China). From birth to the end of the experiment, farm staff and two researchers monitored and recorded calf health status daily via Leiweishi cameras (Guangzhou Leiweishi Security Technology Co., Ltd., Guangzhou, Guangdong, China, 2003), including body temperature, respiratory rate, and physical condition. Respiratory rate (no./min) was measured at three time points (8:00 a.m., 2:00 p.m., and 8:00 p.m.) using a stopwatch, and the average of these three measurements was recorded as the daily respiratory rate. Overall, the management procedures and feeding methods were similar for both groups. No calves developed diarrhea, pneumonia, or other clinical diseases during the experiment.

**Figure 1:**
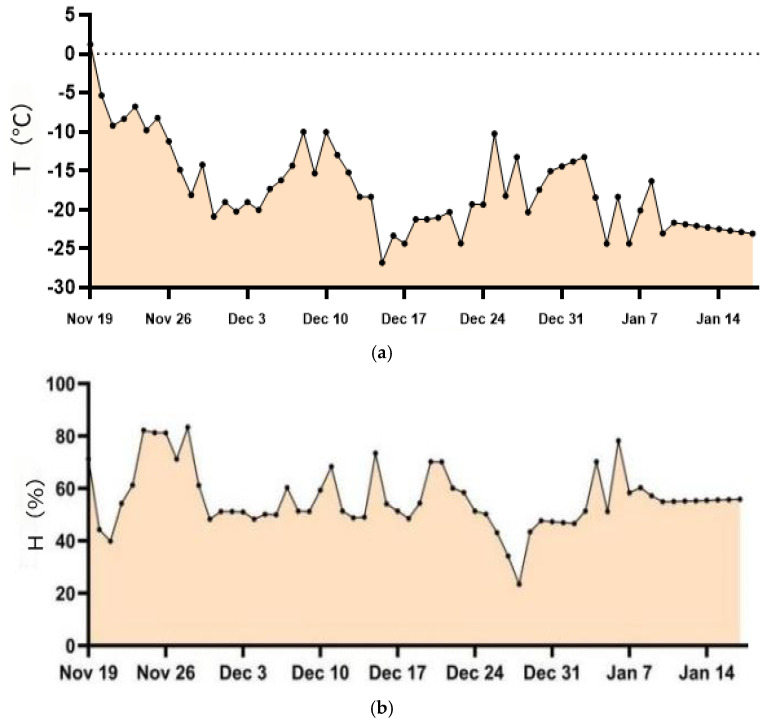
The curves of temperature and humidity changes inside and outside the barn during the experimental period (**a**,**b**), where T represents temperature and H represents humidity. (**a**) The curve of temperature change outside the barn during the experimental period. (**b**) Line chart of humidity change outside the barn during the experimental period.

A total of 60 days of experimental data were collected for 20 calves, recording the highest average outdoor temperature of 1.21 °C, average relative humidity of 71.30%, and wind speed of 9.34 km/h, and the lowest average outdoor temperature reached −26 °C, with relative humidity of 73% and wind speed of 8.46 km/h, as shown in Figure 1a,b, where T denotes temperature and H denotes humidity. The temperature and humidity inside the barn were kept constant, with an average temperature of 5 °C and an average relative humidity of 75%.

### 2.2. Determination of Blood Immune, Antioxidant Indicators, and Blood Metabolome

On days 1, 30, and 60, blood was collected pre-feeding via jugular vein into anticoagulant-free tubes and centrifuged (3500 r/min, 15 min, 4 °C, Yancheng Anxin Test Instrument Co., Ltd., Yancheng, Jiangsu, China, 2021) to isolate serum. Serum antioxidant (T-AOC, GSH-Px, and MDA) and immune (IgA, IgG, and IgM) indices were measured using ELISA kits. The ELISA kits used in this study were bovine-specific commercial kits (YX-21842B, Shanghai Youxuan Biotechnology, Shanghai, China, 2023).

During the experiment, blood samples were collected via jugular venipuncture into vacuum blood collection tubes without anticoagulant. Serum was separated by centrifugation at 4 °C and stored in liquid nitrogen at −80 °C. Raw data were converted into the mzXML format using the MScoder program from Proteovisard, Madison, WI, USA. Mass spectrum peak extraction and quality control were performed using XCMS software (version 4.8.0, Bioconductor, The Scripps Research Institute, La Jolla, CA, USA), and adduct ion annotation was conducted via CAMERA (version 1.5.1, Bioconductor, The Scripps Research Institute, La Jolla, CA, USA) for metabolite recognition and identification. Metabolite identification and annotation were carried out using the metaX program (version 2.0, R package, GitHub, Beijing, China): MS^1^ data were matched with public databases for identification, while MS^2^ data were identified by comparison with an in-house standard database, and the identified metabolites were further annotated using the HMDB and KEGG databases. Finally, metabolite quantification and differential metabolite screening were performed using metaX software (version 2.0, R package, Beijing, China).

For sample preparation, a 20 μL aliquot of the biological sample was added to 120 μL of 50% methanol, vortexed thoroughly, and incubated at room temperature for approximately 10 min to extract metabolites. The extract was stored at −20 °C overnight for protein precipitation, followed by centrifugation at 4000 r/min for 20 min, and the supernatant containing metabolites was transferred to a 96-well plate. A 10 μL aliquot of each diluted sample was mixed equally to prepare QC samples, and all metabolic samples were stored at −80 °C prior to analysis.

The raw mass spectrometry data were converted into a readable format using MSConvert software (version 3.0, ProteoWizard, Palo Alto, CA, USA) from ProteoWizard. Peak extraction and quality control were then performed using XCMS software (version 4.8.0, Bioconductor, The Scripps Research Institute, La Jolla, CA, USA), and adduct ion annotation of the extracted compounds was conducted with CAMERA (version 1.5.1, Bioconductor, The Scripps Research Institute, La Jolla, CA, USA). Metabolite identification was carried out using the metaX software (version 2.0, R package, Beijing, China). Database matching was performed based on primary mass spectrometry data, or identification was achieved by matching secondary mass spectrometry data against an in-house standard database. The tentatively identified metabolites were further annotated using databases including HMDB and KEGG to illustrate their physicochemical properties and biological functions. Finally, metabolite quantification and differential metabolite screening were analyzed using the metaX program.

### 2.3. Data Preprocessing

Environmental, behavioral, and physiological indicators were measured at three time points to determine cold stress values via a cold stress evaluation model. Non-normally distributed variables (respiratory frequency, respiratory rate, and behavioral characteristics) necessitated the use of a generalized linear mixed model (GLMM) to analyze relationships between environmental factors, behavioral traits, physiological parameters, and cold stress severity.

GLMM combines generalized linear models (GLMs) and linear mixed models, offering unique advantages for repeated measures analysis. Parameter estimation via maximum likelihood enhanced model validity, confirming the comprehensive model’s effectiveness in evaluating dairy cow cold stress.

The general form of the linear mixed model is as follows:

y=Xβ+Zγ+ε

where *y* is an *n* × 1 vector representing observed values, *X* is an *n* × *p* matrix representing fixed-effect independent variables, *β* is a *p* × 1 vector representing fixed-effect parameter vectors, *Z* is an *n* × *q* matrix representing random-effect vectors, *γ* is a *q* × 1 vector representing the effects of random factors, and *ε* is an *n* × 1 vector representing residuals (random errors). Let the linear predictor η represent the combination of fixed and random effects (excluding random errors), i.e.,

η=Xβ+Zγ

Let g(⋅) denote the link function, which connects the linear predictor to the label. If h(⋅) is the inverse function of g(⋅), then

g(E(y))=η, E(y)=H(η)

Thus, the GLMM is expressed as:

y=h(η)+ε

The best-fitting model was selected based on the Akaike Information Criterion (AIC) and Bayesian Information Criterion (BIC). The significance level was set to (α=0.05), corresponding to a confidence level of 1 − 0.05 = 0.95, i.e., a 95% confidence interval.

This study investigates the impact of cold stress intensity on various indicators of dairy calves, making the GLMM suitable for interpretation. By analyzing the interrelationships among indicators in the evaluation system, a hierarchical structure was established. The outdoor cold stress evaluation system for calves consists of four layers: target layer, constraint layer, criterion layer, and solution layer.

Constructing the judgment matrix: Determine the relative importance of indicators at all levels and establish the judgment matrix for each level of indicators. Compare the importance of each indicator, *A_i_* and *A_j_*, and assign values accordingly.

The judgment matrix has the following properties: $a_{ij}=1,\;a_{ij}=1/a_{ji},\;a_{ii}=1$.

The judgment matrix adopts the 1–9 scaling method proposed by Satty, as shown in Fu et al. [10]. Based on this method, we conducted a questionnaire survey on 20 experts in dairy cow research (8 experts in dairy cow nutrition and feed science, 5 experts in dairy cow production, and 7 ranch staff) and 8 farmers. The invited experts hold extensive professional knowledge and practical experience, such as disease prevention, breeding management, nutritional management and intelligent management, etc., across all stages, from calves to heifers. They were able to assess the significance of environmental, physiological, and behavioral factors on the growth of outdoor calves, and provided the ranking of their respective indicator layers based on the researcher’s elaboration of the experimental design. The enterprise sector included senior executives (general managers, directors, etc.) and practitioners (nutritionists, technicians, etc.), and the university sector covered doctoral supervisors and senior researchers. To ensure data authenticity and score validity, it was necessary that the experts fully understood the constructed indicator system. Invitations to participate were sent promptly upon candidate confirmation, after screening for a clear research background, a clarified scoring purpose, and indicator connotations. Anonymous opinion solicitation avoided expert interference and ensured scoring objectivity.

Calculate the Weight Vector

For each judgment matrix of the secondary or tertiary indicator system, normalize each column. Then, sum the normalized columns by row to obtain a column vector. Normalize this column vector again to derive the eigenvector corresponding to the judgment matrix of the indicator system. This eigenvector serves as the weight vector for each indicator in the system. Calculate the maximum eigenvalue of the judgment matrix to conduct a consistency check.

Hierarchical Single Sorting and Consistency Check

The steps for the consistency check are generally as follows:

(1) Calculate the consistency index (*CI*)

$$CI=\frac{\lambda_{\max}-n}{n-1}$$

The corresponding average random consistency index (RI) is shown in Table 1.

(2) Calculate the consistency ratio (CR)

$$CI=\frac{CR}{RI}$$

When CR < 0.10, the consistency of the judgment matrix is acceptable; otherwise, the judgment matrix needs to be revised.

(3) Hierarchical Total Sorting and Consistency Check. The total sorting weights should be synthesized top-down from the weights under single criteria.

### 2.4. Construction of Multilevel Fuzzy Comprehensive Evaluation Model

Setting the Evaluation Set

**Figure 2:**
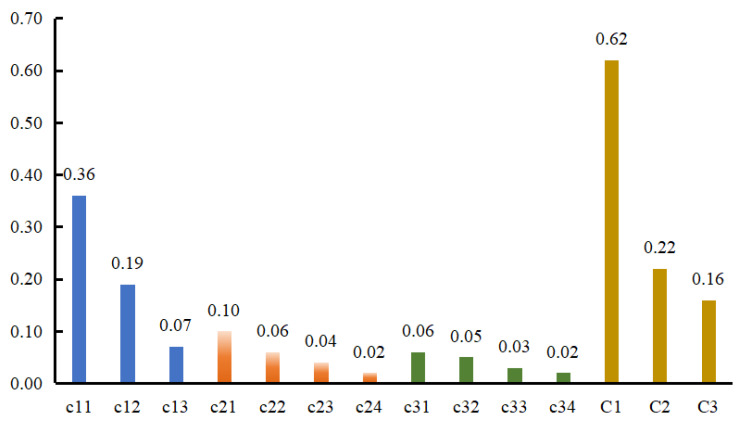
The weight values of evaluation indicators at each level, where C1–C3 are the weight values of first-level evaluation indicators, and c11–c34 are the weight values of second-level evaluation indicators.

Due to the different evaluation values of each indicator factor, they often form different degrees. The set composed of different judgments is called the evaluation set. Based on the evaluation index system for cow cold stress (Figure 2), the evaluation indicator factor set and evaluation set are determined, as shown in Figure 2.

(1) Set the first-level evaluation indicator factor set B = (B1, B2, B3), and the second-level evaluation indicator factor sets B1 = (b11, b12, b13), B2 = (b21, b22, b23, b24), B3 = (b31, b32, b33, b34).
(2) Set the evaluation set V_j_ = (V_1_, V_2_, …, V_j_), where j = 1, 2, …, 5. V_1_ to V_5_ represent five levels of cold stress: none, mild, moderate, severe, and extreme.

### 2.5. Statistical Analysis

After organizing the data using Excel, the independent samples *t*-test was performed for difference comparison analysis using SPSS 22.0 statistical software. A value of *p* < 0.05 indicates a significant difference, *p* < 0.01 indicates an extremely significant difference, and *p* > 0.05 indicates no significant difference.

## 3. Results

### 3.1. Judgment Matrices and Weight Vectors of Indicators

The 1–9 scaling method was used to score the importance of indicator factors at each level. The weight vectors of indicators at all levels were obtained through “calculating weight vectors” in data processing and analysis. Table 2 shows the judgment matrices and weight vectors of indicators at each level based on the scores of experts and ranch staff.

Evaluation of Cold Stress Degree

The degree of cold stress can be divided into five levels: no cold stress, mild cold stress, moderate cold stress, severe cold stress, and extreme cold stress. Taking arbitrary data of a calf on November 9 as an example, the indicators were: temperature 1.21 °C, relative humidity 71.30%, wind speed 9.34 m/s, body weight 36.7 kg, body height 76 cm, body diagonal length 55 cm, chest circumference 80 cm, lying time 1290.39 min, standing time 149.61 min, respiratory rate (RR) 34 times/min, and urination 2 times. The weights of corresponding second-level indicator factors (b11, b12, b13, b21, b22, b23, b24, b31, b32, b33, b34) in the cold stress evaluation model are: C1 = [0.36, 0.19, 0.07], C2 = [0.10, 0.06, 0.04, 0.02], C3 = [0.06, 0.05, 0.03, 0.02]. The fuzzy evaluation matrices E1, E2, E3 of first-level indicators are obtained through expert scoring:

$$E1=\left(\begin{array}{ccccc} 0 & 1 & 0 & 0 & 0\\ 0 & 0.9 & 0.1 & 0 & 0\\ 1 & 0 & 0 & 0 & 0 \end{array}\right)E2=\left(\begin{array}{ccccc} 1 & 0 & 0 & 0 & 0\\ 0.8 & 0.2 & 0 & 0 & 0\\ 0.7 & 0.3 & 0 & 0 & 0\\ 1 & 0 & 0 & 0 & 0 \end{array}\right)E3=\left(\begin{array}{ccccc} 0.8 & 0.2 & 0 & 0 & 0\\ 0.9 & 0.1 & 0 & 0 & 0\\ 0.6 & 0.4 & 0 & 0 & 0\\ 1 & 0 & 0 & 0 & 0 \end{array}\right)$$

The weights of corresponding second-level indicators in the cold stress evaluation model are: W1=(0.39, 0.11, 0.05), W2=(0.14,0.05,0.03,0.13), W3=(0.06, 0.03, 0.02, 0.01). The comprehensive evaluation results of environmental factors, physiological factors, and behavioral factors are as follows:

B1=W1∗E1=(0.05, 0.49, 0.01, 0, 0)

B2=W2∗E2=(0.33, 0.02, 0, 0, 0)

Next, the second-level fuzzy judgment $B={\left(B_{1},B_{2},B_{3}\right)}^{T}$ set is used as the fuzzy relation matrix for the first-level comprehensive judgment. The first-level index weights are as follows:

The comprehensive judgment vector is calculated as:

V=W∗B=(0.15, 0.27, 0.01, 0, 0)

After normalization (0.35, 0.64, 0.13, 0, 0), the maximum membership degree of 0.64 is obtained. According to the maximum membership principle, the comprehensive evaluation of cold stress belongs to the second level, so the comprehensive evaluation result is “mild cold stress”.

The Kendall’s rank correlation coefficients were 0.830 (*p* < 0.01), −0.830 (*p* < 0.01), 0.311 (*p* < 0.05), −0.685 (*p* < 0.01), and 0.797 (*p* < 0.01), for lying time, standing time, respiratory rate (RR), malondialdehyde (MDA), and total antioxidant capacity (T-AOC) of calves at three time points, indicating that lying time, RR, and T-AOC increased with the increase in THI degree, while standing time and MDA decreased with the increase in THI degree.

### 3.2. Fixed-Effect Results Derived from GLMM

#### 3.2.1. Association Analysis Between Standing Time and THI

The fixed-effect term for standing time was statistically significant (F = 898.359, *p* < 0.001), with an intercept of 181.162 (*p* < 0.001, as shown in Table 3). The main effects of THI 1 and THI 2 on standing time were both significant (*p* < 0.001), with coefficient values of 398.133 and 246.781, respectively. That is, for every 1-degree increase in THI, the standing time corresponding to THI 1 and THI 2 increased by 398.133 and 246.781 min, respectively.

#### 3.2.2. Association Analysis Between Respiratory Rate and THI

The fixed-effect term for respiratory rate was statistically significant (F = 3.624, *p* = 0.04), with an intercept of 38.1 (*p* < 0.001, as shown in Table 3). The main effect of THI 1 on respiratory rate was significant (*p* = 0.013), while that of THI 2 was not significant (*p* = 0.069), with coefficient values of −5.1 and −4.3, respectively. That is, for every 1-degree increase in THI, the respiratory rate corresponding to THI 1 decreased by 5.1 times.

#### 3.2.3. Association Analysis Between THI and MDA

The fixed-effect term for MDA was statistically significant (F = 19.3, *p* < 0.001), with an intercept of 4.289 (*p* < 0.001, as shown in Table 3). Both THI 1 and THI 2 had significant main effects on MDA (*p* < 0.001, *p* = 0.069), with coefficient values of 2.892 and 1.372, respectively. That is, for every 1-degree increase in THI, MDA corresponding to THI 1 and THI 2 increased by 2.892 and 1.372, respectively.

#### 3.2.4. Association Analysis Between T-AOC and THI

The fixed-effect term for T-AOC was statistically significant (F = 44.598, *p* < 0.001), with an intercept of 26.414 (*p* < 0.001, as shown in Table 3). Both THI 1 and THI 2 had significant main effects on T-AOC (both *p* < 0.001), with coefficient values of −10.345 and −6.88, respectively. That is, for every 1-degree increase in THI, T-AOC corresponding to THI 1 and THI 2 decreased by 10.345 and 6.88, respectively.

#### 3.2.5. Association Analysis Between Growth Indicators and THI

The fixed-effect term of body diagonal length was statistically significant (F = 142.182, *p* < 0.001), with an intercept of 49.506 (*p* = 0.036, as shown in Table 3). The main effect of THI 1 on body diagonal length was significant (*p* = 0.001), while that of THI 2 on respiratory rate was not significant (*p* = 0.077), with coefficient values of 28.208 and 8.302, respectively. That is, for every 1-degree increase in THI, the body diagonal length corresponding to THI 1 increased by 28.208 cm.

### 3.3. Effects of Cold Environment on Growth Performance and Behavioral Characteristics of Suckling Calves

#### 3.3.1. Effects of Cold Environment on Body Weight and Behavioral of Suckling Calves

The indoor environment maintained an average temperature of 5 °C. The outdoor group had an average respiratory rate of 33–35 breaths/min, compared to 43–45 breaths/min in the indoor group. The outdoor group averaged 1.91 urinations per day, while the indoor group had 1.42 urinations per day. The average lying time was 1027.12 min/24 h for the outdoor group and 918.68 min/24 h for the indoor group, as shown in Table 4.

As shown in Table 5, there was no significant difference in body weight between the two groups of calves at the beginning of the experiment (*p* > 0.05), and no significant changes were observed in the average daily gain (ADG) and body weight from day 1 to 30 (*p* > 0.05). However, from day 30 to 60, the body weight and ADG of the outdoor group were significantly higher than those of the indoor group (*p* < 0.01). Although there was no significant difference in pelleted feed intake, the intake of the outdoor group was higher than that of the indoor group. Throughout the 60-day experimental period, the body weight and average daily gain of the outdoor group were consistently significantly higher than those of the indoor group (*p* < 5.01).

#### 3.3.2. Effects of Cold Environment on Body Size Indicators of Suckling Calves

As shown in Table 5, there was no significant difference in body size indicators between the two groups of calves at the beginning of the experiment (*p* > 0.05). By day 60, the chest circumference and cannon circumference of the outdoor group were significantly larger than those of the indoor group (*p* < 0.01). In addition, from day 1 to 60, the increments of body height and chest circumference in the outdoor group were significantly higher than those in the indoor group (*p* < 0.05).

#### 3.3.3. Effects of Cold Environment on Immune Function of Suckling Calves

As shown in Table 6, there was no significant difference in the contents of IgA, IgG, and IgM in the serum of calves between the outdoor and indoor groups from day 1 to 60 (*p* > 0.05).

#### 3.3.4. Effects of Cold Environment on Antioxidant Function of Suckling Calves

As shown in Table 6, at the beginning of the experiment, there was no significant difference in the contents of T-AOC, GSH-Px, and MDA in the serum of calves between the indoor and outdoor groups (*p* > 0.05). On day 60 of the experiment, the contents of T-AOC and MDA in the serum of the outdoor group were higher, and the content of GSH-Px was lower than those of the indoor group, but the differences were not significant (*p* > 0.05).

#### 3.3.5. Effects of Cold Environment on Blood Metabolism of Suckling Calves

**Figure 3:**
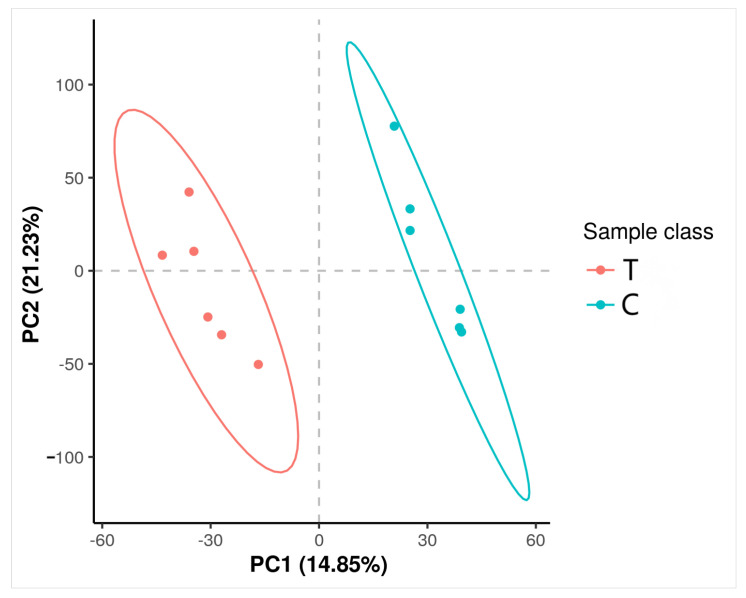
PLS-DA scores of plasma samples from the outdoor group and the indoor group.

PLS-DA Analysis of Blood Metabolites. In this study, partial least-squares discriminant analysis (PLS-DA) was used to construct a relationship model between metabolite expression profiles and sample classification, as shown in Figure 3. The model showed strong prediction performance and stability, with an R2Y value of 0.9864 and a Q2 value of 0.5387—both indicators exceeded the threshold of 0.5 and approached 1. As shown in Figure 3, there were significant differences in plasma metabolite profiles between the outdoor and indoor groups.

Analysis of Differential Blood Metabolites. Differential metabolites were screened based on the following criteria: (1) VIP value ≥ 1; (2) fold-change (FC) ratio ≥1.5 or ≤1/1.5; (3) *p*-value ≤ 0.05. Metabolites that simultaneously met the above three criteria were determined as differential metabolites. Through the comprehensive analysis of the external and internal group datasets, the top 20 metabolites with the most significant differences were finally screened, among which 11 metabolites showed up-regulated expression, and 9 showed down-regulated expression, as shown in Table 7.

**Figure 4:**
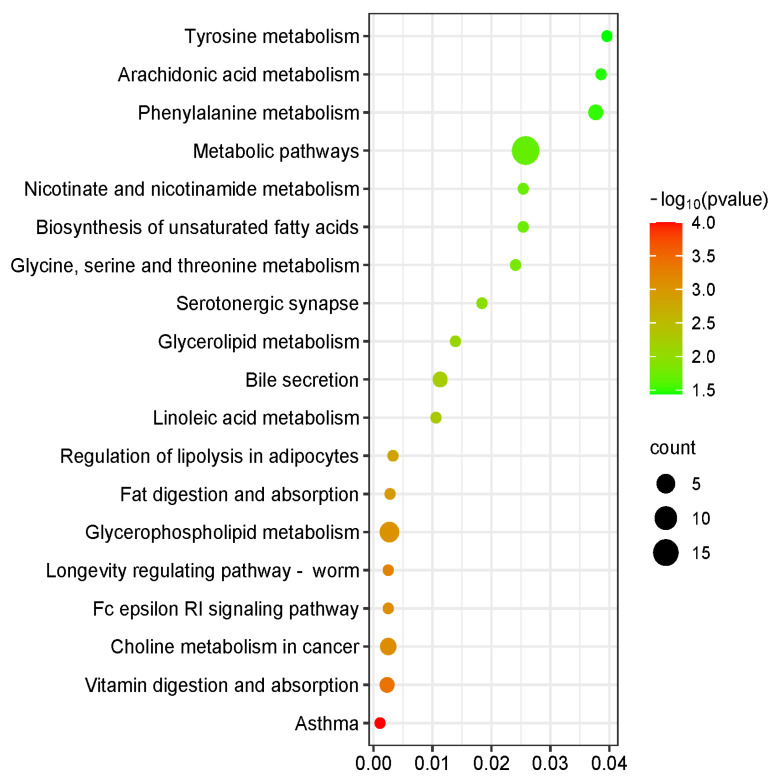
Pathway enrichment analysis of differentially expressed metabolites. Note: Insulin resistance, Choline metabolism in cancer, Asthma, Glycerophospholipid metabolism, Glycerolipid metabolism, Linoleic acid metabolism, Longevity regulating pathway-worm, Bile secretion, Vitamin digestion and absorption, Fc epsilon RI signaling pathway, Glycine, serine and threonine metabolism, Phenylalanine metabolism, Tyrosine metabolism, Arachidonic acid metabolism, Biosynthesis of unsaturated fatty acids, Nicotinate and nicotinamide metabolism, Fat digestion and absorption, Regulation of lipolysis in adipocytes, Serotonergic synapse, Metabolic pathways, Serotonergic synapse.

The KEGG database was used to analyze the pathways enriched by differential metabolites, and the top 20 KEGG pathways with the smallest Q-values were used for mapping. The vertical axis represents metabolic pathways, the horizontal axis represents the enrichment factor, the size of the circle represents the quantity, and the redder the color, the smaller the Q-value. A total of 20 metabolic pathways were obtained through pathway enrichment analysis. As shown in Figure 4, the screened metabolic pathways were: choline metabolism in cancer, asthma, glycerophospholipid metabolism, glycerolipid metabolism, linoleic acid metabolism, longevity regulating pathway-worm, bile secretion and vitamin digestion and absorption, Fc epsilon RI receptor signaling pathway difference, fat digestion, absorption and fat decomposition regulation, glycine, serine and threonine metabolism, tyrosine metabolism, phenylalanine metabolism, arachidonic acid metabolism, biosynthesis of unsaturated fatty acids, nicotinic acid and nicotinamide metabolism, metabolic pathways, and serotonergic synapse. According to the analysis of the KEGG database (Figure 4), the choline metabolism in cancer, asthma, glycerophospholipid metabolism, glycerolipid metabolism, linoleic acid metabolism, longevity regulating pathway-worm, bile secretion and vitamin digestion and absorption, Fc epsilon RI receptor signaling pathway difference, fat digestion and absorption, and fat decomposition regulation in the serum of dairy calves in the outdoor group and indoor group were extremely significant (*p* < 0.01), and glycine, serine and threonine metabolism, tyrosine metabolism, phenylalanine metabolism, arachidonic acid metabolism, biosynthesis of unsaturated fatty acids, nicotinic acid and nicotinamide metabolism, metabolic pathways, and serotonergic synapse were significantly different (*p* > 0.05).

## 4. Discussion

This study focused on several environmental indicators such as temperature, relative humidity, and wind speed in dairy cow sheds. To our knowledge, this study is the first to comprehensively evaluate physiological, behavioral, and environmental indicators in relation to outdoor calf rearing in cold environments, as well as to conduct a variance analysis of biochemical indicators such as blood. Through research on indoor and outdoor environmental indicators, it was found that these factors significantly affect calf growth. Therefore, by integrating physiological factors (body weight, body diagonal length, chest circumference, and body height) and behavioral factors (lying time, standing time, respiratory rate, and urination frequency), a cold stress evaluation method based on AHP-FCE (analytic hierarchy process–fuzzy comprehensive evaluation) was proposed. This method constructs a 2 × 2 comparison judgment matrix as the basis of AHP, evaluated by experienced dairy cow experts. Additionally, the membership degree theory in FCE is a reliable approach to transform human experience and knowledge into mathematical models. Based on the above analysis, this study converts evidence from the literature and northern winter environmental quality standards into a fuzzy evaluation model, which more reasonably reflects the cold stress status of calves.

Behavioral changes in dairy calves are responses to external stress, used to regulate physiological imbalances. When dairy calves are in a state of cold stress for a long time, they maintain body temperature through corresponding behaviors. Additionally, behavioral characteristics are also an external manifestation of dairy cows’ health status. In cold environments, calves often curl up to conserve heat, leading to increased lying time in outdoor-reared suckling calves. Meanwhile, cold stimulation of the hypothalamic–pituitary axis reduces antidiuretic hormone secretion, thereby increasing urination frequency. Furthermore, accelerated metabolism caused by cold leads to more frequent defecation [11]. Studies on the seasonal physiological changes in calves in Sichuan found that respiratory rate decreases in cold environments because deep breathing helps reduce heat loss during respiration [11]. This study used a Generalized Linear Mixed Model (GLMM) to statistically analyze the repeated measurements of THI and behavioral indicators of calves inside and outside the shed. The results showed significant correlations between indoor and outdoor data (all *p* < 0.001), and suckling calves raised in cold environments had increased lying time, urination and defecation frequencies, and decreased respiratory rate. Therefore, this study proposes a calf cold stress evaluation method based on AHP-FCE, which integrates physiological factors such as body weight and body diagonal length, and behavioral factors such as lying time and respiratory rate. The model is established by experts constructing a 2 × 2 comparison judgment matrix and combining the membership degree theory of fuzzy comprehensive evaluation, which can reasonably reflect the cold stress status of calves.

Research on the production performance and developmental status of suckling calves in cold environments remains limited. As core indicators for evaluating calf growth performance, changes in body weight, average daily gain (ADG), and body size are particularly important. Existing studies have shown that cold environments prompt animals to increase energy consumption to maintain basal metabolic functions, which is often a key factor leading to weight loss [12]. However, this study found a noteworthy phenomenon: calves raised in outdoor cold environments showed more significant advantages in body weight and body size growth compared to those in indoor normal-temperature environments. GLMM analysis showed that for every 1-unit increase in the Temperature-Humidity Index (THI), the body diagonal length corresponding to THI1 increased by 28.208 cm. Data from the early stage (first 30 days) of the study showed that the daily gain and body weight indicators of the cold-exposed group were superior to those of the indoor group. This result may be related to the metabolic regulation mechanism triggered by the cold environment: to cope with cold stress, calves need to enhance metabolic heat production to maintain thermal balance. During this process, the energy provided by regular breastfeeding can no longer meet their growth needs, prompting calves to increase pelleted feed intake. It is worth noting that throughout the experiment, the milk intake of the two groups of calves remained similar, but the pelleted feed consumption of the outdoor group was significantly higher. This finding is consistent with the conclusion of [13] that pelleted feed intake has a significant impact on calf weight gain and body size development. Studies by Roland L et al. [14] also support this view, finding that calves in cold environments not only showed faster weight growth but also had higher pelleted feed consumption. The National Research Council (NRC) [9] and other related studies further confirm that appropriate supplementation of pelleted feed in cold environments can effectively promote the weight gain of suckling calves. The data from the present study showed that after 30 days of pelleted feed supplementation, the body weight and ADG indicators of the outdoor group calves significantly exceeded those of the indoor group, fully indicating that a cold environment combined with reasonable feed management can significantly improve the growth performance of suckling calves.

Immunoglobulin M (IgM) is produced in the initial stage of the antibody response, while Immunoglobulin G (IgG) plays a key role in the systemic immune response and is the main antibody in serum after antigen exposure. Immunoglobulin A (IgA) is the main antibody of mucosal immunity, working together with innate non-specific defense mechanisms to repel pathogens. IgA, IgG, and IgM together constitute key indicators of animal immune function, reflecting their immune capacity. An S An et al. [15] found no significant difference in serum IgG and IgM levels between heifers raised in cold environments and those under non-stress conditions. Similarly, Zhou G C. et al. [16] reported that cold environments had no significant effect on serum IgA, IgG, and IgM levels in sheep. Consistent with these findings, this experiment showed that serum IgA, IgG, and IgM levels in outdoor group calves were not significantly affected, possibly because the calves adapted to the cold environment and had no exposure to foreign bacteria. Chardonnay’s study in Baotou, Inner Mongolia, in X Fu et al. [10] found that the serum GSH-Px activity of Holstein cows during cold stress was significantly lower than that during non-stress periods [17,18]. Lin W M and Meng X K et al. [19,20] observed that with the decrease in environmental temperature, serum T-AOC and MDA levels in calves increased, while GSH-Px activity decreased. However, Yao et al. [21] showed no significant differences in T-AOC, MDA, and GSH-Px levels between lambs raised in cold environments and the non-stress group, which is consistent with the test results [22]. In the present trial, calves raised in cold environments showed increased T-AOC and MDA levels, along with decreased GSH-Px activity. GLMM analysis showed that both THI 1 and THI 2 had significant main effects on MDA (*p* < 0.001 and *p* = 0.069), with regression coefficients of 2.892 and 1.372, respectively. That is, for every 1-unit increase in THI, MDA levels corresponding to THI 1 and THI 2 increased by 2.892 and 1.372, respectively. Additionally, the fixed effect of T-AOC was statistically significant (F = 44.598, *p* < 0.001), with an intercept of 26.414 (*p* < 0.001). Both THI 1 and THI 2 had significant main effects on T-AOC (both *p* < 0.001), with regression coefficients of −10.345 and −6.88, indicating that for every 1-unit increase in THI, T-AOC levels corresponding to THI 1 and THI 2 decreased by 10.345 and 6.88, respectively. However, no significant changes were observed in antioxidant indicators of the indoor group. Overall, the impact of a cold environment on the immune and antioxidant capacity of suckling calves is relatively limited.

Impact of Cold Environment on Amino Acid Metabolism. Under cold stress, the body enhances amino acid metabolism, leading to increased production of metabolites such as phenylalanine, citrulline, glycine, glutamic acid, and L-carnosine in the blood and liver. Glycine is an α-amino acid whose amino group is connected to the α-carbon adjacent to the carboxyl group. Amino acids contain amino and carboxyl functional groups, distinguished by their unique side chains (R groups), and are basic organic compounds involved in protein biosynthesis. Serine and threonine are also α-amino acids with the same characteristic structure, where the amino group is connected to the carbon atom adjacent to the carboxyl group. Among the 20 amino acids used in protein synthesis, these amino acids play key roles in maintaining various physiological processes, especially under cold stress. This study found significant differences in the metabolism of glycine, serine, and threonine. Additionally, downstream metabolites of the phenylalanine pathway, particularly phenylacetylglycine, were found to help increase ATP production. In addition to energy production, intermediates of glycolysis (one of the main metabolic pathways) play a key role in synthesizing other compounds that integrate carbohydrate, protein, and fat metabolism [23]. This enhanced metabolic activity may support increased protein synthesis, helping calves maintain energy balance and resist the effects of cold.

Impact of Cold Environment on Fatty Acid Metabolism. 3-Methylindole (also known as skatole) is produced in the small intestine by the anaerobic microbial decomposition of undigested proteins, especially tryptophan. After further degradation of tryptophan, skatole is absorbed into the circulatory system through the intestinal wall, partially metabolized in the liver and excreted in the urine, and the rest is stored in adipose and muscle tissues. Elevated skatole levels can cause acute bovine pulmonary edema and emphysema in ruminants [24]. In this experiment, the relative abundance of 3-methylindole decreased, indicating that calves raised in cold environments may have a reduced risk of pulmonary edema and emphysema. Additionally, the relative abundance of diacylglycerol phosphate 60:15 increased in this study. Natural fats mainly contain unsaturated fatty acids, usually with one (such as oleic acid) or two double bonds (such as linoleic acid). As an essential unsaturated fatty acid, linoleic acid plays a key role in regulating blood lipid metabolism. Triacylglycerols (TAGs), including derivatives such as triacylglycerol 66:21, are key energy storage molecules, and linoleic acid is a major component of these TAGs [25]. KEGG pathway analysis showed significant differences in the biosynthesis of unsaturated fatty acids and linoleic acid metabolism in this trial. The up-regulation of linoleic acid and hexadecanoic acid, combined with the decrease in 3-methylindole, indicates positive effects on lipid metabolism and glucose homeostasis. These changes may help maintain calf health, improve energy storage, and enhance cold resistance.

## 5. Conclusions

This study elucidated the actual effects of cold outdoor environments on calf growth and development. The results showed that even in cold outdoor environments, calves did not experience growth retardation or health impairment; on the contrary, cold outdoor environments were beneficial to calf growth. Meanwhile, environmental factors were the most critical factors affecting calf cold stress, and the Temperature–Humidity Index (THI) significantly correlated with multiple physiological and behavioral indicators in the calves. Differential metabolites were mainly enriched in energy metabolism and linoleic acid metabolism pathways. These findings provide an important theoretical basis for optimizing the outdoor feeding management model of calves in winter and also offer practical references for the subsequent regulation of calf cold stress and the improvement of feeding schemes.

## Figures and Tables

**Table 1:** Values of RI for *n*-order Matrix.

*n*	1	2	3	4	5	6	7	8	9	10
RI	0	0	0.58	0.90	1.12	1.24	1.32	1.41	1.45	1.49

Note: *n* represented the order of the judgment matrix, and RI the consistency index.

**Table 2:** Calculating weights of dairy cow indicators at all levels based on expert scores and AHP-FCE.

	b11	b12	b13	b21	b22	b23	b24	b31	b32	b33	b34	B1	B2	B3
Expert														
b11	1	2	5											
b12	0.50	1	3											
b13	0.25	0.33	1											
b21				1	2	3	3							
b22				0.50	1	2	3							
b23				0.33	0.50	1	2							
b24				0.33	0.33	0.50	1							
b31								1	2	2	2			
b32								0.50	1	3	4			
b33								0.50	0.33	1	2			
b34								0.50	0.25	0.50	1			
B1												1	4	3
B2												0.25	1	2
B3												0.33	0.50	1
*n*	3			4				4				3		
wi or wii	0.36	0.19	0.07	0.10	0.06	0.04	0.02	0.06	0.05	0.03	0.02	0.62	0.22	0.16
CR2	0.003			0.026				0.090				0.094		
farmer														
b11	1	5	7											
b12	0.20	1	3											
b13	0.14	0.33	1											
b21				1	3	5	2							
b22				0.33	1	2	3							
b23				0.20	0.50	1	3							
b24				0.50	0.33	0.33	1							
b31								1	3	3	4			
b32								0.33	1	2	3			
b33								0.33	0.50	1	2			
b34								0.25	0.33	0.50	1			
B1												1	2	4
B2												0.50	1	4
B3												0.25	0.25	1
*n*	3			4				4				3		
wi or wii	0.39	0.11	0.05	0.14	0.05	0.03	0.13	0.06	0.03	0.02	0.01	0.54	0.35	0.11
CR2	0.003			0.026				0.090				0.094		

Note: b11, b12, b13 were the three environmental factors; b21, b22, b23, b24 were the four physiological factors; b31, b32, b33, b34 were the four behavioral factors; B1, B2, B3 were the first level factors; *n* denoted the number factors contained in each indicator set; wi or wii was the weight values of evaluation indicators at the first or the second level; and CR2 was the consistency ratio for the second level.

**Table 3:** GLMM fixed-effect results of behavioral factors, immune factors, physiological factors and THI.

Dependent Variable	Model	Coefficient	SE	t	*p*	α	AIC	BIC
Standing Time	Intercept	181.162	0.8858	29.82	<0.01	0.05	261.65	264.494
	THI = 6.86	398.133	9.4661	42.059	<0.01	0.05		
	THI = 11.95	246.781	14.1562	17.433	<0.01	0.05		
Respiratory Rate	Intercept	38.1	1.6155	23.583	<0.01	0.05	170.198	173.042
	THI = 6.86	−5.1	1.9117	−2.668	0.013	0.05		
	THI = 11.95	−4.3	2.2718	−1.893	0.069	0.05		
MDA	Intercept	4.289	0.3051	14.06	<0.01	0.05	90.189	93.034
	THI = 6.86	2.892	0.4663	6.202	<0.01	0.05		
	THI = 11.95	1.372	0.4194	3.272	<0.01	0.05		
T-AOC	Intercept	26.414	0.8858	29.82	<0.01	0.05	124.74	127.58
	THI = 6.86	−10.345	1.0956	−9.443	<0.01	0.05		
	THI = 11.95	−6.88	0.9598	−7.168	<0.01	0.05		
Body Straight Length	Intercept	49.506	22.3234	2.218	0.036	0.05	148.31	150.98
	THI = 6.86	28.208	7.1639	3.937	<0.01	0.05		
	THI = 11.95	8.302	4.5076	1.842	0.077	0.05		

Note: SE—standard error, t—t statistics, α—significance level, AIC—Akaike information criterion, BIC—Bayesian information criterion, MDA—malondialdehyde content, and T-AOC—total antioxidant capacity.

**Table 4:** Changes in behaviors of calves.

	Indoor Group	Outdoor Group	SEM	*p*
Lying down time (min/24 h)	918.68 ^b^	1027.12 ^a^	10.28	<0.01
Walking/standing time (min/24 h)	521.32 ^a^	412.88 ^b^	10.29	<0.01
Frequency of urination (once/24 h)	1.42 ^b^	1.91 ^a^	0.471	0.040
Frequency of defecation (once/24 h)	1.47 ^b^	1.83 ^a^	0.125	<0.01
Respiratory rate (once/min)	44.86 ^a^	34.51 ^b^	0.618	<0.01

Note: Within the same row, values with different lowercase letters (a, b) indicate significant difference (*p* < 0.05). *p* < 0.01, highly significant difference. SEM, standard error of the mean.

**Table 5:** Effects of cold environment on body weight, feed intake and growth of Holstein suckling calves.

Items	Indoor Group	Outdoor Group	SEM	*p*
Day 1–30				
Initial weight (kg)	35.28	35.23	1.259	0.969
End weight (kg)	57.69	56.15	1.251	0.234
Average daily weight gain (kg/d)	0.76	0.66	1.509	0.343
Day 30–60				
Initial weight (kg)	57.69	56.15	1.251	0.234
End weight (kg)	78.86 ^b^	89.11 ^a^	1.673	0.004
Average daily weight gain (kg/d)	0.71 ^b^	1.22 ^a^	1.963	<0.01
Average pelleted feed intake (g)	189.915	254.14	36.948	0.063
Day 1–60				
Initial weight (kg)	35.28	35.23	1.259	0.969
End weight (kg)	78.86 ^b^	89.11 ^a^	1.673	<0.01
Average daily weight gain (kg/d)	0.73 ^b^	0.89 ^a^	0.029	<0.01
Average pelleted feed intake (kg)	189.915	254.14	36.948	0.063
Day 1				
Height (cm)	76.2	75.7	1.757	0.779
Body diagonal length (cm)	61.1	58.7	1.275	0.708
Chest circumference (cm)	81.5	82.8	1.022	0.220
Circumference of pipe (cm)	12.3	12.1	0.658	0.767
Day 30				
Height (cm)	81.60	83.45	1.378	0.172
Body diagonal length (cm)	70.61 ^a^	65.2 ^b^	1.795	<0.01
Chest circumference (cm)	96.30	105.2	0.681	0.472
Circumference of pipe (cm)	13.2 ^b^	14 ^a^	0.468	<0.01
Day 60				
Height (cm)	87.1 ^b^	90.4 ^a^	0.891	<0.01
Body diagonal length (cm)	79.4 ^b^	81.9 ^a^	1.795	<0.01
Chest circumference (cm)	105.9 ^b^	111.5 ^a^	0.949	<0.01
Circumference of pipe (cm)	13.70 ^b^	16.44 ^a^	0.367	<0.01
Day 1–60				
Body size increase				
Height (cm)	12 ^b^	17 ^a^	1.731	<0.01
Body diagonal length (cm)	18.3 ^b^	25.3 ^a^	1.904	<0.01
Chest circumference (cm)	24.4 ^b^	29.5 ^a^	1.989	<0.01
Circumference of pipe (cm)	1.4 ^b^	4.34 ^a^	0.016	<0.01

Note: Within the same row, values with different lowercase letters (a, b) indicate significant difference (*p* < 0.05). *p* < 0.01, highly significant difference. SEM, standard error of the mean.

**Table 6:** Effects of cold environment on immune function and antioxidant function in Holstein suckling calves.

Items	Indoor Group	Outdoor Group	SEM	*p*
1 d				
IgA (μg/mL)	3145.78	2564.73	263.42	0.070
IgG (ng/mL)	495.97	401.90	92.11	0.365
IgM (μg/mL)	1708.41	1985.61	256.29	0.340
30 d				
IgA (μg/mL)	3117.61	2441.48	358.43	0.108
IgG (ng/mL)	590.06	550.82	65.62	0.582
IgM (μg/mL)	2628.29	2831.72	464.91	0.684
60 d				
IgA (μg/mL)	3765.56	3573.05	618.57	0.771
IgG (ng/mL)	568.02	435.24	57.91	0.084
IgM (μg/mL)	2368.87	2389.51	405.41	0.962
1 d				
T-AOC (U/mL)	27.9	27.88	1.66	0.990
GSH-Px (U/L)	99.01	97.03	5.07	0.718
MDA (nmol/mL)	4.45	4.42	0.91	0.977
60 d				
T-AOC (U/mL)	14.60	16.19	1.541	0.361
GSH-Px (U/L)	157.33	151.07	3.544	0.152
MDA (nmol/mL)	7.74	7.81	0.976	0.950

Note: SEM represents the standard error of the mean. IgA, IgG, IgM, T-AOC, GSH-Px, and MDA are abbreviations for Immunoglobulin A, Immunoglobulin G, Immunoglobulin M, total antioxidant capacity, glutathione peroxidase, and malondialdehyde.

**Table 7:** Differential metabolites in blood from the outdoor group and the indoor group.

Distribution of Differential Metabolites	Ratio	*p* Value	VIP	Regulation
3-Methylindole	0.29	0.003	3.285	down
1-Methoxy-1H-indole-3-carboxaldehyde	0.26	0.010	3.529	down
Phenaceturic acid	0.48	0.008	2.005	down
Inosine	0.17	0.022	3.415	down
Guanosine	0.17	0.006	3.391	down
2,2′-Methylene-bis(6-tert-butyl-4 methylphenol)	0.21	0.033	3.851	down
LysoPC 16:1	0.67	0.026	1.735	down
Taurolithocholic acid sulfate	0.47	0.013	1.769	down
HemiBMP 54:12; HemiBMP (18:4/18:4/18:4)	1.75	0.002	1.511	up
HemiBMP 60:15; HemiBMP (20:5/20:5)	1.86	0.013	1.471	up
Hydroquinone	2.86	<0.01	2.843	up
Alpha-Ketooctanoic acid	1.56	0.016	2.307	up
1-Methoxy-1H-indole-3-carboxaldehyde	0.26	0.010	3.529	down
DL-Indole-3-lactic acid	1.73	0.017	2.088	up
Dodecanedioic acid	1.89	0.004	2.417	up
Hexadecanedioic acid	4.86	<0.01	3.828	up
Linoleic acid	1.59	0.004	1.997	up
15,16-DiHODE	1.53	0.004	1.316	up
8alpha-8-Hydroxy-12-oxo-13-abieten-18-oic acid	1.69	0.029	1.742	up
9-Oxo-11-(3-pentyl-2-oxiranyl)-10E-undecenoic acid	3.05	<0.01	3.376	up

Note: VIP represents variable importance in projection.

## Data Availability

Data and procedure will be made available upon request to the corresponding authors. The role of the funding sponsor includes the conceptualization, funding acquisition, supervision, writing, review and editing, and he decided to publish the research results.
